# Rapid upwards spread of non-native plants in mountains across continents

**DOI:** 10.1038/s41559-022-01979-6

**Published:** 2023-01-26

**Authors:** Evelin Iseli, Chelsea Chisholm, Jonathan Lenoir, Sylvia Haider, Tim Seipel, Agustina Barros, Anna L. Hargreaves, Paul Kardol, Jonas J. Lembrechts, Keith McDougall, Irfan Rashid, Sabine B. Rumpf, José Ramón Arévalo, Lohengrin Cavieres, Curtis Daehler, Pervaiz A. Dar, Bryan Endress, Gabi Jakobs, Alejandra Jiménez, Christoph Küffer, Maritza Mihoc, Ann Milbau, John W. Morgan, Bridgett J. Naylor, Aníbal Pauchard, Amanda Ratier Backes, Zafar A. Reshi, Lisa J. Rew, Damiano Righetti, James M. Shannon, Graciela Valencia, Neville Walsh, Genevieve T. Wright, Jake M. Alexander

**Affiliations:** 1 Institute of Integrative Biology, ETH Zurich, Zurich, Switzerland; 2 UMR CNRS 7058, Ecologie et Dynamique des Systèmes Anthropisés, Université de Picardie Jules Verne, Amiens, France; 3 Institute of Biology / Geobotany and Botanical Garden, Martin Luther University Halle-Wittenberg, Halle, Germany; 4 German Centre for Integrative Biodiversity Research (iDiv), Leipzig, Germany; 5 Department of Land Resources and Environmental Sciences, Montana State University, Bozeman, MT USA; 6 Instituto Argentino de Nivología, Glaciología y Ciencias Ambientales, CONICET, Mendoza, Argentina; 7 Department of Biology, McGill University, Montreal, Quebec Canada; 8 Department of Forest Ecology and Management, Swedish University of Agricultural Sciences, Umeå, Sweden; 9 Research Group Plants and Ecosystems, University of Antwerp, Wilrijk, Belgium; 10 Department of Planning, Industry and Environment, Queanbeyan, New South Wales Australia; 11 Department of Botany, University of Kashmir, Srinagar, India; 12 Department of Environmental Sciences, University of Basel, Basel, Switzerland; 13 Department of Botany, Ecology and Plant Physiology, Faculty of Sciences, University of La Laguna, La Laguna, Spain; 14 Departamento de Botánica, Facultad de Ciencias Naturales y Oceanográficas, Universidad de Concepción, Concepción, Chile; 15 Instituto de Ecología y Biodiversidad, Santiago, Chile; 16 School of Life Sciences, University of Hawaii at Manoa, Honolulu, HI USA; 17 Department of Botany, Amar Singh College, Srinagar, India; 18 Eastern Oregon Agricultural Research Center, Oregon State University, La Grande, OR USA; 19 Atelierschule Zurich, Zurich, Switzerland; 20 Laboratorio de Invasiones Biológicas, Facultad de Ciencias Forestales, Universidad de Concepción, Concepción, Chile; 21 Eastern Switzerland University of Applied Sciences, Rapperswil, Switzerland; 22 Department of Sustainable Environment and Nature Policy, Antwerp, Belgium; 23 Research Centre for Applied Alpine Ecology, Department of Ecology, Environment and Evolution, La Trobe University, Bundoora, Victoria Australia; 24 USDA Forest Service, Pacific Northwest Research Station, Forestry and Range Sciences Lab, La Grande, OR USA; 25 Centre for Ocean Life, Technical University of Denmark, Kgs Lyngby, Denmark; 26 Swiss Federal Research Institute WSL, Birmensdorf, Switzerland; 27 Royal Botanic Gardens, South Yarra, Victoria Australia

**Keywords:** Invasive species, Climate-change ecology, Biodiversity

## Abstract

High-elevation ecosystems are among the few ecosystems worldwide that are not yet heavily invaded by non-native plants. This is expected to change as species expand their range limits upwards to fill their climatic niches and respond to ongoing anthropogenic disturbances. Yet, whether and how quickly these changes are happening has only been assessed in a few isolated cases. Starting in 2007, we conducted repeated surveys of non-native plant distributions along mountain roads in 11 regions from 5 continents. We show that over a 5- to 10-year period, the number of non-native species increased on average by approximately 16% per decade across regions. The direction and magnitude of upper range limit shifts depended on elevation across all regions. Supported by a null-model approach accounting for range changes expected by chance alone, we found greater than expected upward shifts at lower/mid elevations in at least seven regions. After accounting for elevation dependence, significant average upward shifts were detected in a further three regions (revealing evidence for upward shifts in 10 of 11 regions). Together, our results show that mountain environments are becoming increasingly exposed to biological invasions, emphasizing the need to monitor and prevent potential biosecurity issues emerging in high-elevation ecosystems.

## Abstract (web summary)

The authors resurveyed a previously sampled set of mountain transects on five continents, showing that the ranges of non-native plant species have shifted upslope in most locations in just 5–10 years.

## Main

Species’ distributions are being reshuffled across the globe at unprecedented rates^1–5^. These redistributions are particularly visible in mountain ecosystems, where species can cross large environmental gradients across relatively short distances^6,7^. While many native species are on the move in mountains in response to changing climate^8–10^, mountain ecosystems have so far experienced comparatively few invasions by non-native species^11,12^. Possible reasons for this include less-intense human activity and disturbance at high elevation or that few non-native species can thrive in high-elevation conditions^11,13–16^. However, this situation is changing with climate warming and increasing human pressures at high elevation^11,15,17^. For instance, road verges in mountain ecosystems are already known to provide more suitable conditions for non-native plants at higher elevations than would be expected without disturbances^14,18,19^ and, within individual regions, some non-native plants are expanding their ranges upward faster than native species^15,20^.

Understanding how quickly the richness and elevational distribution of non-native species is changing in mountain regions is important if we are to react appropriately to the challenges (for example, conservation issues) and opportunities (for example, ecosystem services) they may pose. The few regional studies assessing temporal changes of non-native plant distributions in mountains have revealed either no average expansion over a decade (in Europe^21^) or upward range expansions over several decades to a century (in Europe^15^, Hawaii^22^ and California^20^). However, we have so far lacked the necessary time-series data to assess how consistent range limit changes are worldwide or whether (and ultimately why) they differ among regions with contrasting climates, land use histories and species introductions.

**Fig. 1:**
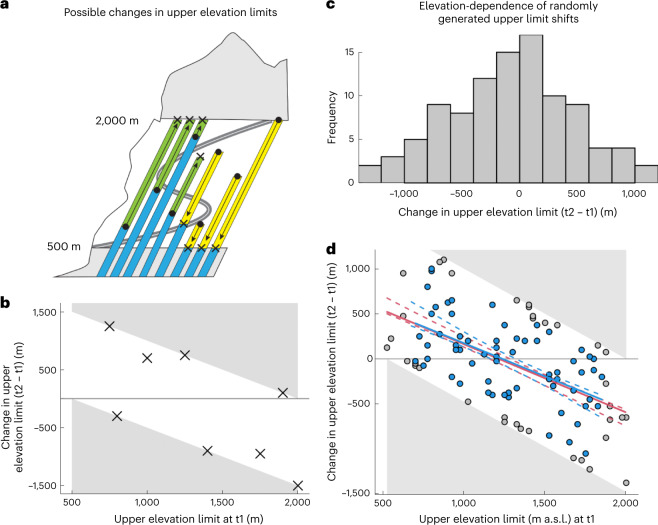
A null-model approach to explaining species’ range limit shifts across an elevational gradient. **a**, Possible changes in observed upper elevation limit for eight species (bars) shifting their upper limits upwards (green portions of bars: expansions) or downwards (yellow portions of bars: contractions) along an elevational gradient from time point 1 (t1; points) to time point 2 (t2; crosses). The species’ distributions are surveyed at sites along a hypothetical mountain road 500–2,000 m above sea level (a.s.l.) (within the unshaded region), although all of them also occur at lower elevations below the surveyed elevational window. **b**, The observed range shifts of the upper elevation limit for the eight example species (crosses) are plotted against their upper elevation limits at t1. The grey triangles delimit the boundaries of the surveyed elevational gradient (compare grey shaded regions in **a**) and hence represent ‘non-observable’ shifts in elevation limits. **c**, Histogram of upper limit shifts for 100 species, generated randomly within the constraints imposed by the boundaries of the observed elevational gradient (compare **b**). **d**, The boundaries of the observed elevational gradient give rise to the null expectation of a negative relationship between species’ upper limits during t1 and their shift at the upper elevation limit due to chance alone. The red line indicates the fitted relationship and dotted red lines indicate 95% CIs for the expected relationship based on 1,000 resamples (with replacement) of the 100 species’ elevational limits at t1, accounting for the geometric constraint. Excluding species occurring in the top and bottom 10% of the gradient either at t1 or t2 (remaining species coloured in blue) results in the fitted relationship depicted by the blue line. The dotted blue lines indicate 95% CIs calculated as described above but excluding species falling in the top and bottom 10% of the gradient.

Studies have shown that most non-native plant species in mountains are initially introduced at low elevation and from there spread upwards to fill their climatic niche^13^. As a result, species that have been introduced for a longer period tend to reach progressively higher elevations^23–25^. This leads to the prediction that, over time and under stable climatic conditions, upward range shifts will be most pronounced for non-native species that are found initially at low elevation and spread upwards to fill their climatic niche, and less evident for species already found at high elevation, although climate warming could contribute to accelerate upward shifts also at high elevation^20^. Studies of climate-induced range shifts in native plant species have repeatedly found a similar negative relationship between species’ initial upper range limits and the magnitude of their upper range limit expansion (Fig. 1). Contrasting mechanisms have been proposed to explain this pattern, including: (1) stronger responses of warm-adapted (low elevation) species to climate warming^15,26^; (2) changes in climatic water balance or biotic interactions driving downward migrations at higher elevation^27,28^; (3) longer growing seasons and correspondingly greater opportunities for dispersal and spread at lower elevation^29^; (4) broader environmental tolerances of high-elevation species, necessitating smaller absolute range shifts to track changing climate^10^; and (5) greater microsite variation at high elevation, buffering the need for range shifts^7,30^.

Here, we propose and examine an alternative hypothesis, that larger upward range limit shifts among species previously found at lower elevations are expected by chance alone. This null expectation applies to any range observations made across a finite elevational gradient, for two reasons. Firstly, the statistical phenomenon of regression toward the mean describes how unusually large or small values of a variable on average tend to be followed by measurements closer to the mean, giving rise to a negative correlation between the initial value of a variable and the change in paired measurements of that variable over time (refs. ^31,32^; Supplementary Methods). In the context of range limits, this means that under the assumption that range limit changes occur at random, species with originally especially low or high initial upper range limits are expected to have range limits closer to the mean elevation in the next time step. This leads to a null expectation of a negative relationship between species’ initial upper range limit and its change over time.

Secondly, the geometric constraint imposed by the boundaries of a finite gradient, for example from the valley bottom to the mountain summit, means that upward shifts cannot be observed for species already present at the highest surveyed elevation and vice versa for downward shifts of species previously limited to the lowest elevation, leading to a mid-domain effect in terms of degrees of freedom that is well recognized in the scientific literature^33^. A common approach to mitigate this problem has been to remove species already reaching either end of the studied gradient (for example, refs. ^10,29^). However, the geometric constraint on the magnitude and direction of observable range shifts (compare refs. ^33,34^) applies across the whole gradient and not only to species whose ranges already reach the top or bottom of the elevational survey. As Fig. 1 demonstrates, large upward shifts can only be observed for species originally restricted to the lower part of the gradient, while large downward shifts can only be observed for species originally restricted to the upper part. Therefore, even if range shifts were completely random within a set of species (no average upward or downward shifts), we would expect a negative relationship between the initial elevational distribution of range limits and range limit shifts, simply due to constraints on the observable magnitude and direction of range shifts along elevational gradients (Fig. 1). Excluding species occurring at the top and bottom of the gradient does not remove this effect (Fig. 1d). Consequently, before seeking ecological explanations for observed patterns of range dynamics across environmental gradients, these patterns should be evaluated against appropriate null models^27,33,35^. However, very few studies of climate-related range shifts use null models to interpret patterns of range limit shifts or to fully account for geometric constraints^10,35^.

Here, we analysed data from a standardized survey of non-native plant distributions (developed by the Mountain Invasion Research Network (MIREN); www.mountaininvasions.org, ref. ^36^) to quantify and compare temporal patterns of invasions in mountains across the globe. We surveyed elevational gradients along mountain roads and adjacent seminatural vegetation in 11 mountain ranges from 5 continents, ranging from 68° N (Norway) to 37° S (Australia). Our data includes nearly 15,000 observations of 616 non-native plant species from 651 sampling transects resurveyed every 5 years across a 5-year (five regions) or 10-year (six regions) period. We analysed temporal changes in the richness and upper elevational limits of non-native plant species, introducing a null-model approach to interpret patterns of range limit shifts across elevational gradients after accounting for geometric constraints and compare these patterns across regions. Specifically, we ask: (1) has the richness of non-native plant species in mountains increased during the last decade? and (2) are the upper elevation limits of non-native species moving upslope and, if so, are upslope shifts occurring faster than expected at lower elevations?

## Results and discussion

**Fig. 2:**
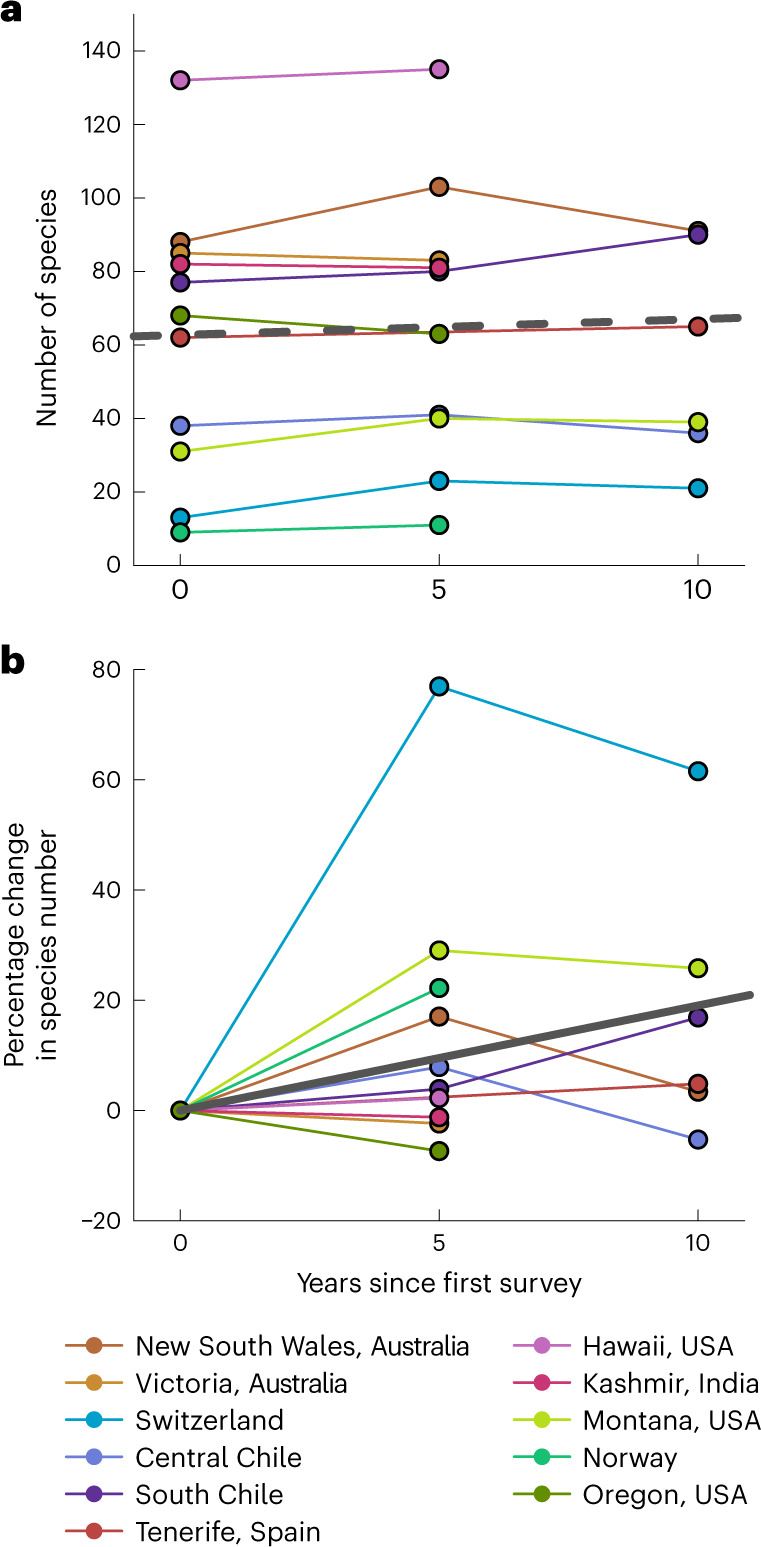
Temporal changes of non-native plant species in 11 mountain regions. **a**,**b**, Total species richness (**a**) and percentage change in species richness (**b**) over the 10-year sampling period. Solid and dashed heavy grey lines indicate significant and non-significant fits, respectively, from mixed-effects models including all non-native species occurring at least twice in a region (see text). Not all regions were sampled in all years.

After first excluding species occurring only once in a region to reduce possible bias caused by very infrequent species (reducing the total number of non-native species from 616 to 480), the total non-native plant species richness increased in 7 out of 11 (64%) regions over the whole study period. Increases ranged from 2 to 15 species depending on the region but changes were non-monotonic in three of the five regions that were sampled three times (Fig. 2a). Four regions (Oregon (United States), Central Chile, Victoria (Australia) and Kashmir (India)) experienced a small net loss of non-native species and, in Central Chile, the loss occurred after initial gains in species richness during the first 5-year interval. Overall, this resulted in a close to significant increase in non-native species richness across all regions of 0.46 ± 0.23 (mean ± s.e.) species per year (*χ*^2^ = 3.190, *P* = 0.074). Expressed as percentage changes (Fig. 2b), this corresponded to a significant net increase of 1.56 ± 0.57% in non-native species richness per year (model slope ± s.e., *χ*^2^ = 6.822, *P* = 0.009) or ~16% over a decade, due to proportionally larger increases in regions with fewer non-native species.

Temporal trends in non-native plant species richness within individual regions may be partially obscured by environmental conditions in the year of the survey. For example, unusually dry conditions in 2017 may explain the decline in non-native species richness detected during that year in Switzerland, while the increase in non-native species richness in New South Wales (Australia) in 2012 was probably caused by much higher rainfall in 2011. However, independent of whether regional changes in non-native species richness are influenced by climate anomalies or survey artifacts (such as observer effects), by pooling observations from multiple independent regions located across the globe, we could detect an overall trend of non-native plant richness generally increasing in mountain regions over time, even over a short 5- to 10-year period.

**Fig. 3:**
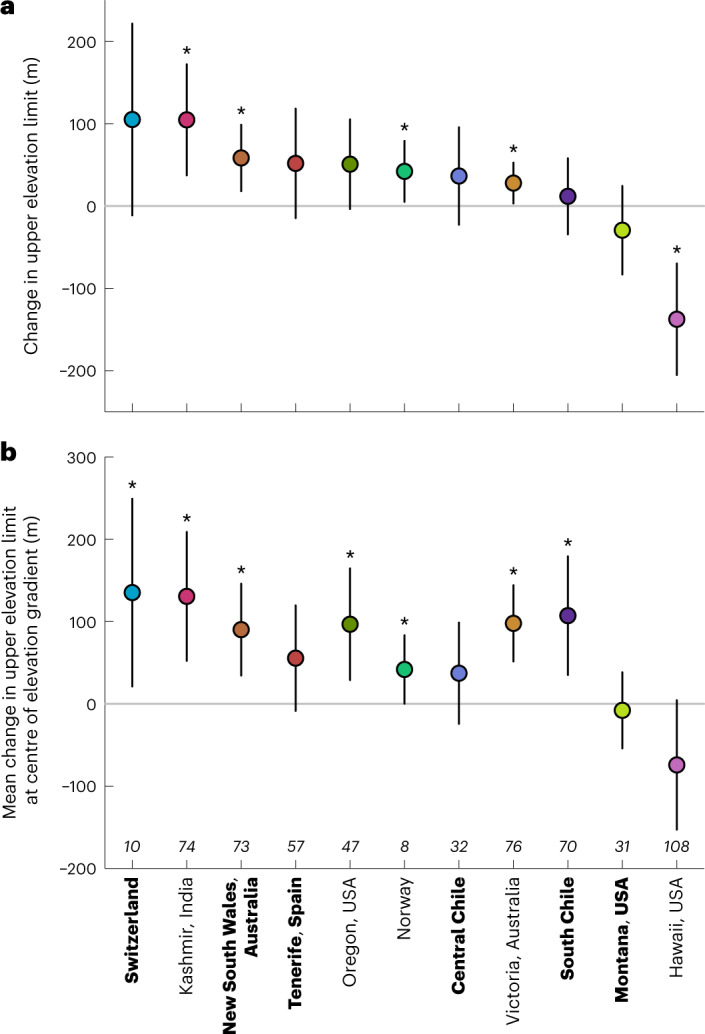
Observed changes in species’ upper elevational limits (±95% CIs). Both panels show mean shifts between the first and last survey in each region, estimated from linear models that weight species by their total frequency of occurrence in both years and are fitted to data from each region separately (for mean annual shifts see Supplementary Fig. 1). **a**,**b**, Results of intercept-only models (grand mean shifts per region) (**a**) and results of models that correct for elevation by including species’ initial elevation limit during the first survey as a linear predictor (**b**). Specifically, estimates in **b** correspond to the predicted mean shift in elevation limits when evaluated at the median elevation within a given region; values >0 therefore indicate that average shifts are upslope across most of the elevational gradient (compare Fig. 4). Regions are ordered by effect size in **a**, with labels in regular and bold typeface indicating regions with 5- or 10-year survey intervals, respectively. Estimates that differ significantly from zero are indicated by *. Numbers in italics describe the sample size; colours correspond to the same labels as in Fig. 2.

On average, non-native plant species shifted their upper elevational limits (defined as the 90th percentile of their observed elevations of occurrence) upslope in all regions except Montana and Hawaii (United States). Mean upslope shifts between the first and last survey were significant in four regions (and close to significant (*P* < 0.1) in a further two regions; intercept-only linear models on unstandardized elevation, with observations weighted according to species’ overall frequency of occurrence; Fig. 3a and Supplementary Table 1). We found the same pattern for mean annual upslope shifts (Supplementary Fig. 1a and Supplementary Table 2). These results were also robust to applying stricter filters excluding less common species to examine the influence of low species frequency in some of the regions: retaining only species with >5 (filter 1) and >10 (filter 2) occurrences per region over all years resulted in identical trends with upslope shifts in all regions, except Hawaii and Montana (United States), and significant upslope changes in three regions. As the overall occurrence of non-native plant species was very low in Norway, the upslope shift was no longer significant for filter 1 and the region was excluded for analysis with filter 2, as fewer than five species remained in the dataset (Supplementary Table 3). When only retaining species with >10 occurrences per region and year (filter 3), three regions (Norway, Switzerland and Central Chile) had fewer than five species remaining and were therefore excluded. The range shift direction did not change for the remaining eight regions (with the same three regions showing significant upslope shifts), except for South Chile, where changes were always non-significant (Supplementary Table 3).

Overall changes in upper elevation limits across regions (standardized range limit shift is 0.07 ± 0.04 in an intercept-only linear mixed-effects model (LMM) with region as a random effect: *t* = 1.77, d.f. = 9.43, *P* = 0.109) were obscured by large variation in the magnitude and direction of shifts along gradients and among regions. Regional variation in the magnitude of range shifts was partly due to differences in the extent of the studied elevational gradients and therefore in the average distances between surveyed transects (ranging from 17 m in Norway and Victoria to 76 m in Kashmir) with Kashmir, Hawaii, Tenerife and Central Chile all having relatively large average distances of >50 m between transects (Supplementary Table 4). In regions with greater distances between surveyed plots we tend to both fail to detect small shifts and potentially to register small shifts as larger than they actually are. This effect may be amplified if species are mostly found along a single road per region, as average distances between transects along a single road will exceed the average distance between transects within regions as a whole. For example, the mean distance between adjacent transects in Hawaii along any given road is 156 m and many species (32%) were only recorded from a single road, which might explain the large downward average upper limit shift in this region. Consequently, large mean range shifts must be interpreted with caution for regions with large average distances between transects.

**Fig. 4:**
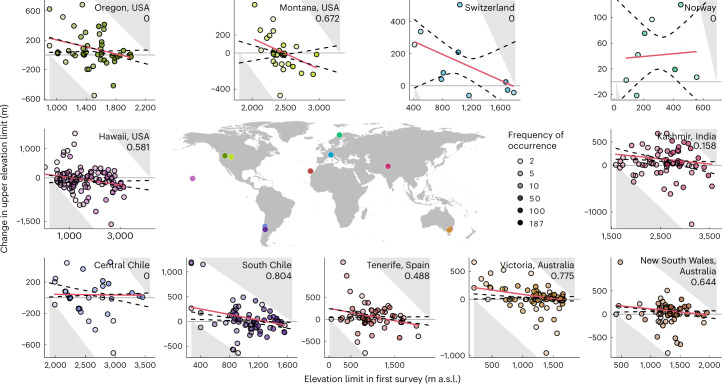
Null-model tests of elevation-dependent shifts in upper elevation limit of non-native plant species in 11 mountain regions. Each point shows the change in upper elevation limit (90th quantile of elevational distribution) of a single species, as a function of its limit in the first survey (darker shading of points corresponds to greater total log(frequency of occurrence) of a species in both surveys, ranging from *n* = 2 (40 species in 10 regions) to *n* = 187 *(Hypochaeris radicata* in Victoria, Australia)). Red regression lines are fitted relationships for observed range limit shifts, weighted by species’ frequency of occurrence. Grey triangles indicate shifts that could not have been observed on the basis of the elevational extent of the field survey (compare Fig. 1); dashed lines are 95% CIs for the expected null relationship between initial upper elevation limit and change in upper elevation limit after accounting for this constraint (Methods; Fig. 1). The proportion of fitted values that fall above or below the CIs in each region are indicated in the top-right of each panel, with non-zero values indicating a significant deviation from the null expectation. Colours correspond to the same labels as in Fig. 2.

Across regions, and as predicted, the upper range limits of non-native plants shifted upslope at low elevations with linearly decreasing magnitude across the elevational gradient, with slight average downslope shifts at high elevations in some regions (LMM of range shifts on initial elevation limit, weighted by species’ frequencies of occurrence: *F*_1,579.89_ = 54.82, *P* < 0.001; Fig. 4). This relationship was significant in six regions when regressions were fitted within individual regions (Supplementary Table 6). On average, upslope shifts at low elevation tended to outweigh downslope shifts at high elevation. To account for these effects of elevation on the average upper range limit shifts, we evaluated the range limit shift at the median of each elevational gradient based on the fitted values of linear regressions in each region. Retaining all species present at least twice per region, upper range limit shifts between the first and last survey were significantly upslope in seven regions (Fig. 3b and for annual shifts see Supplementary Fig. 1b). Although still upslope, range limit shifts were no longer significant for Norway (filter 1) and for Switzerland (filter 2), when excluding less common species. Excluding species occurring fewer than ten times per region and year resulted in non-significant shifts at the midpoint for South Chile and Oregon (Supplementary Table 5; regions with fewer than five species remaining after filtering were excluded from the analysis). This might be due to the low sample size in some regions (Switzerland and Norway) or also reflect the expectation that more widespread species will tend to have been introduced for longer and so are more likely to be in equilibrium with their climatic limits^24^. To conclude, accounting for the elevation dependence of species’ shifts at the upper range limit strengthened our inference that non-native plant species have rapidly expanded their ranges upslope in mountains globally.

Previous studies of both non-native and native species have sought ecological explanations for similar elevation-dependent range shifts to those that we describe here^10,15,26,28,29,37^. However, the bounded nature of survey gradients means that a negative relationship is the default expectation and range shift patterns should therefore be compared against an appropriate null model before they can be interpreted ecologically^35^. Our null-model approach (Methods; Fig. 1) revealed that the observed negative relationships between initial upper elevation limits and range limit shifts fell outside the 95% confidence intervals (CIs) of null expectations in seven regions (both Australian regions, South Chile, Tenerife, Hawaii, Kashmir and Montana; Fig. 4). In all seven cases, observed relationships fell above the CIs of expected relationships in the lower part of the elevational gradients (or in the middle part of the gradient in Kashmir), indicating that species initially restricted to lower (or middle) elevations spread upwards significantly more than expected by chance alone. Greater downward shifts than expected by chance were also observed at high elevation in Tenerife and Montana (Fig. 4). Alternative null models (differing in the vectors of initial elevation limits used to derive expected relationships; Methods) supported an additional significantly greater upward range shift at low elevation than expected by chance in Oregon (Supplementary Figs. 2 and 3). Support for greater downward shifts than expected by chance also differed, depending on the null model used (zero to three regions; Fig. 4 and Supplementary Figs. 2 and 3).

Non-native plant species distributions along elevational gradients tend to display a nested pattern, with species with small ranges mainly restricted to lower elevation sites, where human population density and corresponding opportunities for introduction and establishment are greatest^13,15^. Greater upward shifts in elevation limits for species previously restricted to lower or middle elevations are therefore consistent with a scenario of ongoing dispersal, in which many of these species have only recently been introduced to lower elevation sites. On average, we would not expect recently introduced species to be in equilibrium with their climatic limits but rather to be in a phase of active range expansion (climatic niche filling), in contrast to species that have already established at higher elevations and are closer to their cold limit. Consistent with this hypothesis, recently introduced non-native species in Tenerife tend to have narrower elevation ranges than older introductions^24^, while time since introduction of non-native plant species in Central Europe is positively related to their maximum elevation^23^, elevation range^25^ and to the magnitude of upward range shifts^15^. Conditions at lower elevation might also be more conducive to spread, with longer growing seasons, at least in temperate regions, and greater anthropogenic disturbances and propagule pressure associated with settlements, agriculture and industry. Similar reasons have been proposed to explain the more pronounced elevational shifts and abundance increases of native plants in the European Alps at lower elevation^29^. We hypothesize that a combination of these mechanisms is responsible for the rapid (within 5–10 years) expansion of species’ upper elevation limits that we document in most of our study regions. While climate warming might also have contributed to the upward spread, we did not find a relationship between the average yearly temperature increase between 2000 and 2016 within regions (Supplementary Table 8) and the respective mean regional range limit shifts (linear model of range shifts on average yearly temperature change, *F*_1,9_ = 0.447, *P* < 0.521). Similarly, we did not see a tendency for regions with 10 years of survey data to display greater range shifts over the whole sampling period than regions with only 5 years of survey data, as might be expected if climate change was an important driver. Indeed, significant deviations from null expectations of shifts were already detected over intervals of 5 years in four of five regions with a 10-year time series (Supplementary Table 10). Over the longer term, however, climate warming will extend the suitable elevational range of species to higher elevations, presumably leading to enhanced upslope shifts also in the upper part of the elevational gradients.

In contrast to upward shifts at low elevation, significant deviations from the downward shifts at high elevation predicted by the null model were more scarce. Possible explanations for the few cases of significant downslope shifts might be active removal of non-native species at high elevation in some regions, pushing their upper limits to lower sites^38^, or harsh environmental conditions preceding the final survey (for example, a greater probability of mortality or failed reproduction due to cooler temperatures or severe frost events^21,39^). Downward shifts might also have arisen stochastically, especially if species reaching high elevation tended to be rare and assuming that rarer species are more prone to large range contractions due to stochastic local extinction events^21,27^. Our models partly accounted for this by weighting species on the basis of their frequency of occurrence. In unweighted models, negative relationships between initial elevation limits and range limit shifts were more pronounced in eight regions (Supplementary Table 7), suggesting that stochasticity in colonization and extinction events contribute at least partly to the magnitude of upward and downward shifts.

## Conclusions

In summary, the different lines of evidence we have presented collectively reveal that non-native plant species are expanding their upper elevation limits in 10 out of the 11 surveyed regions. Specifically, upward shifts were chiefly represented by average upper limit changes within regions either before (four regions; Fig. 3a) or after (additional three regions; Fig. 3b) accounting for elevation, or more than expected by chance for species at low/mid elevation (additional three regions; Fig. 4). The only region showing no evidence of significant changes in non-native species’ upper range limits was Central Chile. That these upper range limit changes were observed after just 5 years in five of the regions indicates how rapidly non-native plant species are spreading upwards in mountains around the world, especially along road corridors. By comparison, native plants in the European Alps are moving upslope on average by 28.2 m per decade^40^, which is substantially less than what we report for non-native species (Fig. 3). Roads provide favourable habitat and anthropogenic dispersal routes for many non-native plant species and range expansions along roads are therefore expected to be more rapid than in undisturbed habitats. Non-native plant species spreading away from roads into the surrounding undisturbed vegetation have so far been limited at higher elevation^11,16^ but this might change with increasing anthropogenic disturbance and changing climate. Roadside surveys like ours^36^ might therefore be valuable for the early detection of possible emerging threats to native species and ecosystems. To support such measures, future work should continue to interrogate which species traits promote fastest rates of spread^15,16,29,41,42^, which species have the potential for greatest impacts and which features of high-elevation ecosystems (for example, native community structure, disturbance regimes, rates of climatic or land use changes) are associated with greater levels of invasion^16,43^. Additionally, while negative relationships between historical range positions and range shifts are often observed, our simulations demonstrate that such patterns can also emerge in the absence of biological changes. As time-series data of species’ distribution changes become increasingly available^2^, null models can be powerful tools for evaluating the magnitude and direction of range shifts within given observational constraints and will become crucial to support robust interpretation of range shift dynamics across environmental gradients^35^. Finally, this study suggests that in an era of local and global anthropogenic changes, non-native plant species in mountains will continue to expand upwards regardless of their introduction point. Therefore, any threat posed by non-native species to higher elevation ecosystems will probably increase, making it even more necessary to enact monitoring and management plans in mountain regions around the world.

## Methods

### Data collection

Data were collected by MIREN using a standard protocol^36^. Sampling started in 2007/2008, is repeated every 5 years and is still ongoing. We used data from 11 mountainous regions around the world: the Australian Alps (two regions, New South Wales and Victoria); the Swiss Alps; the Andes (two regions, Central and South Chile); the Montana-Yellowstone National Park (United States); the Blue Mountains (Oregon, United States); Hawaii (United States); Tenerife (Canary Islands, Spain); Kashmir (India); and the Northern Scandes (Norway). For information about geolocation, climate, elevational range and sampling period, see Supplementary Table 4. In each region, three roads were selected (two in Central Chile, four in Hawaii and five in Victoria), all of them open to vehicle traffic for at least part of the year. The bottom of each road was defined as the point below which no major elevational change occurred, while the top was set by the highest point of the road^36^. Each road was evenly stratified by elevation into 20 sampling transects (60 per region, although this varied due to local logistics; Supplementary Table 4), totalling 651 sampling transects. At each location, three 2 × 50 m plots were placed in a T-shape, with one plot parallel to the road and two plots placed adjacent and perpendicular to the first plot, to distinguish between disturbed habitats directly next to the road and more seminatural habitats away from the road (up to 50 and 100 m away from road verges). The two perpendicular plots were only surveyed when there were no impassable barriers such as cliffs and rivers, resulting in unequal numbers of plots per sampling transect (651, 481 and 440 plots at 0, 50 and 100 m from the road, respectively). Sampling was repeated during the peak growing season at 5-year intervals (Supplementary Table 4). The identity of non-native vascular plant species according to the World Flora Online (http://www.worldfloraonline.org) and their abundance (scale 1, 1–10 individuals or ramets; scale 2, 11–100 individuals; and scale 3, >100 individuals) was recorded in every plot. Data from the three plots at each sample transect (elevation) were combined as presence–absence data for each transect for the analyses presented here. The two seminatural plots together accounted for only 35% of the total observations of non-native plant species across years and only 11% of unique observations of non-native plant species within sample transects (observations of species away from but not at the roadside). Further analyses conducted with data separated into road and seminatural plots revealed no consistent differences in average upper limit shifts (Supplementary Table 9). Species not identified to species level were excluded, as were species that were only recorded once in a given region.

### Data analysis

All analyses were carried out in R v.4.0.3, using the lme4 package^44^. The map in Fig. 4 was created on the basis of the R packages sf^45^ and ggmap^46^. All models were checked for compliance with model assumptions. To explore changes in non-native species richness over time, the total number of non-native plant species was calculated for each region and year (*n* = 27 observations of regions/years). Species richness was fitted with LMMs (Gaussian error distribution). Year since first survey (0, 5 or 10 years) was used as a linear predictor and region included as a random intercept term to account for the spatial nestedness of the data. Subsequently, these models were compared to an intercept-only model but still including the same random effect, with a likelihood ratio test. We also fitted LMMs to the percentage change in richness, setting the intercept to zero (percentage change in species richness is always zero in the first year of the survey).

To investigate range expansion of individual non-native species, we calculated for each species the change in upper elevational limit between the first and last time points (over either a 5- or 10-year period). We therefore only retained species present at least once in both the first/last sampling year. Additionally, to assess the influence of less common species on average regional range shifts, we defined three datasets with stricter filters only including species occurring >5 and >10 times per region over all years (filters 1 and 2) and >10 times per region and year (filter 3). The upper elevation limit was defined as the 90th percentile of all species’ occurrences along the elevational gradient. While the 90th percentile approximates the highest occurrence point when species are scarce, it reduces the influence of extreme outlier occurrences when sufficient data points are available to be informative and so provides more conservative estimates of range changes. Range limit changes were quantified as the difference in 90th percentiles between the first (t1) and last time point (t2 or t3) in each region (t2 − t1 or t3 − t1). To facilitate comparisons between regions with very different elevation ranges, elevation was centred and scaled (mean = 0, s.d. = 1) within regions before calculating range limit changes. We first fitted an intercept-only LMM of range limit changes across all species and regions, with region as a random intercept term, to test for overall changes in upper elevation limits across all regions combined. To investigate region-specific patterns, we then fitted intercept-only linear models to unstandardized data from each region separately. In all models, observations were weighted by species’ total frequency of occurrence across the first and last surveys using the ‘weights’ argument within the model formula. We assumed that range-limit estimates for frequently occurring species will be less affected by outlier occurrences than estimates for rare species and therefore gave greater weight to estimates of range shifts for common species.

To test our hypothesis that range expansions are more pronounced at lower elevations, we again fitted an LMM with shift in upper range limit as the response variable and species’ upper elevation limits during the first (t1) survey (standardized within region) as the only fixed effect. Region was used as a random effect (random intercept only) and range shift observations were weighted by species’ total frequency of occurrence as above. We then fitted weighted linear models to data from each region separately (with elevation unstandardized) to test region-specific trends and also fitted unweighted models for comparison. We used the weighted models from each region to estimate mean range shifts of species after accounting for elevation. Specifically, we used the models to predict the mean upper range limit shift and its 95% CI, at the median elevation of species’ upper range limits in the first survey (by setting the predictor variable to the median elevation of the elevational gradient within a given region). If this value is significantly greater than 0, it indicates that average range limit shifts are upslope across most of the elevational gradient in a given region.

Finally, we constructed null models in each region to assess the expected relationship between species’ upper elevation limits in the first survey and shifts in upper elevation limits, under the assumption that observed shifts are entirely stochastic with respect to elevation (observed shifts are placed at random across the elevational gradient). This analysis was performed to account for the fact that the bounded nature of elevational gradients constrains the magnitude and direction of range shifts that are possible to observe at low and high elevation (Fig. 1). Within each region, the observed elevation shifts were randomized across the elevation gradient by randomly drawing new initial elevations (with replacement) from the vector of surveyed elevations (~60 plots per region; Supplementary Table 4). To account for the geometric constraint, the vectors of possible initial elevations for a given iteration of randomized range shift were truncated to values less than or equal to the maximum elevation minus the range shift value in the case of upward range shift values or values greater than or equal to the minimum elevation plus the range shift value in the case of downward range shift values. This procedure was repeated 10,000 times, each time fitting a weighted linear regression of range shift at the upper elevation limit (the response variable) against the initial position of the upper elevation limit (the only predictor variable). Since bootstrap regressions may differ in intercept but not in slope, inference about whether upward shifts differ from the null expectation cannot be based solely on a comparison of the observed and expected slope estimates. For each bootstrap replicate, we therefore retained the fitted values of the regression and used the 10,000 vectors of fitted values to compute 95% CIs around the regression expected under the assumption that range shifts occur at random across the elevational gradient. We compared the observed and expected relationships graphically, concluding that the observed relationship deviated significantly from the null expectation if a non-zero fraction of the regression line fell outside the 95% CIs. We also explored two alternative null models that differed in how the vectors of new initial elevations were defined and therefore in the variation among bootstrap regressions: (1) a less conservative approach (yielding narrower CIs around the expected regression) that used a vector of 200 equally spaced initial elevations, instead of the ~60 elevations that were actually surveyed in each region (results in Supplementary Fig. 2); and (2) a somewhat more conservative approach (yielding wider CIs around the expected regression) that used only the observed initial elevations within each region, that is with a vector length corresponding to the observed number of species (Supplementary Fig. 3).

### Reporting summary

Further information on research design is available in the Nature Portfolio Reporting Summary linked to this article.

## Supplementary information

Supplementary Information Supplementary Figs. 1–3, Tables 1–10, Methods and References.

Reporting Summary

Supplementary Data 1 Environmental data needed for analysis but not published on Zenodo.

Supplementary Data 2 Range shifts of all species in all regions.

Supplementary Code Complete code needed for analysis.

## Data Availability

All datasets generated before 2016 (except two roads in Victoria, Australia) and analysed during the current study are available through the Global Biodiversity Information Facility (GBIF, https://www.gbif.org/publisher/76388ab6-61ca-439a-ab09-e1fe73eb224a) and the complete dataset is available on Zenodo (10.5281/zenodo.7495407) (ref. ^47^). Environmental data and a summary file containing all species and range shifts are provided in Supplementary Data 1 and 2, respectively.

## Code availability

The code used to perform the analysis is available in Supplementary Code.
