# Supplementary material for: Rapid upwards spread of non-native plants in mountains across continents
Source: Nat Ecol Evol. 2023 Jan 26;7(3):405–13. doi: 10.1038/s41559-022-01979-6 (PMC9998268; doi:10.1038/s41559-022-01979-6)
Supplement: Supplementary file 1 — Supplementary Figs. 1–3, Tables 1–10, Methods and References. [file 41559_2022_1979_MOESM1_ESM.pdf]

nature ecology & evolution

Article

https://doi.org/10.1038/s41559-022-01979-6

Rapid upwards spread of non-native plants in mountains across continents

In the format provided by the authors and unedited

Supplemental Information

Supplementary Table 1. Average shifts in upper elevation limits in each region between the first and last survey. Estimates (expressed as m of elevation across the sampling period) are taken from intercept-only linear models fitted to observed shifts in unstandardized upper elevation limits of species in each region separately and weighted by species' frequency of occurrence. Tests were two-sided and no adjustments for multiple comparisons were made. Regions are ordered according to effect size and significant p-values are marked in bold.

	**Estimate ± standard error**	**t-value**	***p***	**N**	**Sampling period (years)**
Switzerland	105.2 ± 51.49	2.04	0.071	10	10
Kashmir, India	104.77 ± 33.86	3.09	**0.003**	74	5
New South Wales, Australia	58.48 ± 20.24	2.89	**0.005**	73	10
Tenerife, Spain	51.86 ± 33.2	1.56	0.124	57	10
Oregon, USA	50.88 ± 27.04	1.88	0.066	47	5
Norway	42.15 ± 15.63	2.7	**0.031**	8	5
Central Chile	36.52 ± 29.01	1.26	0.217	32	10
Victoria, Australia	27.94 ± 12.45	2.25	**0.028**	76	5
South Chile	11.83 ± 23.18	0.51	0.611	70	10
Montana, USA	-29.35 ± 26.32	-1.12	0.274	31	10
Hawaii, USA	-137.52 ± 34.15	-4.03	**< 0.001**	108	5

Supplementary Table 2. Average annual shifts in upper elevation limits in each region. Estimates (expressed as m of elevation per year) are taken from intercept-only linear models fitted to observed shifts in unstandardized upper elevation limits of species in each region separately and weighted by species' frequency of occurrence. Tests were two-sided and no adjustments for multiple comparisons were made. Regions are ordered according to effect size and significant p-values are marked in bold.

	**Estimate ± standard error**	**t-value**	***p***	**N**	**Sampling period (years)**
Kashmir, India	20.96 ± 6.77	3.09	**0.003**	74	5
Switzerland	10.52 ± 5.15	2.04	0.071	10	10
Oregon, USA	10.18 ± 5.41	1.88	0.066	47	5
Norway	8.43 ± 3.13	2.7	**0.031**	8	5
New South Wales, Australia	5.85 ± 2.02	2.89	**0.005**	73	10
Victoria, Australia	5.59 ± 2.49	2.25	**0.028**	76	5
Tenerife, Spain	5.19 ± 3.32	1.56	0.124	57	10
Central Chile	3.65 ± 2.9	1.26	0.217	32	10
South Chile	1.18 ± 2.32	0.51	0.611	70	10
Montana, USA	-2.94 ± 2.63	-1.12	0.274	31	10
Hawaii, USA	-27.5 ± 6.83	-4.03	**< 0.001**	108	5

Supplementary Table 3. Average shifts in upper elevation limits between the first and last survey in each region based on three additional filters for excluding less common species (standard filter >1 occurrence per species and region over all years). Filters used are >5 and >10 occurrences per species and region over all years and >10 occurrences per species, region and year. Estimates (expressed as m of elevation across the sampling period) are taken from intercept-only linear models fitted to observed shifts in unstandardized upper elevation limits of species in each region separately and weighted by species' frequency of occurrence. Tests were two-sided and no adjustments for multiple comparisons were made. P-values of significant range shifts are marked in bold. Models were not fitted for regions with fewer than 5 species after filtering.

	**>5 occurrences/ region over all years**			**>10 occurrences/ region over all years**			**>10 occurrences/ region and year**		
	**Estimate ± standard error**	***p***	**N**	**Estimate ± standard error**	***p***	**N**	**Estimate ± standard error**	***p***	**N**
New South Wales, Australia	60.24 ± 21	**0.006**	66	59.7 ± 23.56	**0.014**	52	74.96 ± 19.33	**0.001**	24
Victoria, Australia	29.32 ± 11.75	**0.016**	55	28.29 ± 11.57	**0.018**	48	31.73 ± 12.02	**0.013**	33
Switzerland	103.24 ± 54.62	0.095	9	109.16 ± 58.48	0.104	8			2
Central Chile	40.64 ± 28.65	0.167	28	56.96 ± 34.14	0.116	16			4
South Chile	7.51 ± 22.74	0.742	58	7.72 ± 23.21	0.741	50	-3.85 ± 21.92	0.862	24
Tenerife, Spain	49.64 ± 38.64	0.206	40	15.86 ± 39.24	0.69	23	104.25 ± 49.53	0.089	6
Hawaii, USA	-150.63 ± 41.21	**0.001**	66	-175.72 ± 53.29	**0.002**	40	-172.75 ± 60.11	**0.018**	10
Kashmir, India	111.05 ± 43.48	**0.014**	47	149.29 ± 49.35	**0.005**	29	184.44 ± 60.44	**0.009**	15
Montana, USA	-27.64 ± 27.33	0.321	28	-29.93 ± 30.17	0.332	23	-59.28 ± 38.48	0.174	7
Norway	44.37 ± 20.68	0.098	5			3			1
Oregon, USA	48.94 ± 28.22	0.091	40	48.38 ± 28.9	0.103	36	40.91 ± 37.89	0.292	22

Supplementary Table 4. Characteristics of the eleven regions of the survey, including: coordinates (latitude and longitude in decimal degrees), the climatic context (Med, Mediterranean; Temp, Temperate; Subtrop, Subtropical), number of roads included in the analysis (and total number of sampling locations ["transects"] per region), the elevational range (and the average distance ± standard deviation between all transects of a region combined), the total number of non-native species in the complete data set over all sampling years (after discarding species not determined to the species level and species occurring less than twice per region over all years), and the years in which regions were surveyed. All regions were surveyed at 5-year intervals, except Tenerife (10 year interval).

	Geolocation (units)	Climate	Roads (transects)	Elevational range (m) (ø distance between transects)	Species richness	Sampling years
New South Wales, Australia	-35.984, 148.402	Med	3(60)	410–2125 (32.36 ± 20.01)	141	2007, 2012, 2017
Victoria, Australia	-37.142, 147.105	Med	5(99)	205–1848 (17.29 ± 15.7)	114	2012, 2017
Switzerland	46.262, 7.54	Temp	3(63)	415–1800 (26.15 ± 18.76)	33	2007, 2012, 2017
Central Chile	-33.349, -70.299	Med	2(31)	1900–3585 (67.4 ± 48.37)	50	2007, 2012, 2017
South Chile	-37.516, -71.61	Temp	3(60)	277–1664 (23.43 ± 17.62)	113	2007, 2012, 2017
Tenerife, Spain	28.241, -16.563	Semiarid	3(56)	13–2310 (50.79 ± 35.84)	89	2008, 2018
Hawaii, USA	20.046, -155.699	Tropical	4(74)	212–4180 (53.68 ± 57.41)	235	2007, 2012
Kashmir, India	33.68, 74.888	Subtrop	3(28)	1590–3644 (75.9 ± 56.74)	114	2012, 2017
Montana, USA	44.795, -110.188	Temp	3(60)	1807–3311 (25.43 ± 27.61)	46	2007, 2012, 2017
Norway	68.263, 17.633	Subarctic	3(60)	14–692 (17.19 ± 19.22)	14	2012, 2017
Oregon, USA	45.273, -117.625	Temp	3(60)	902–2264 (23.9 ± 17.76)	103	2007, 2012

Supplementary Table 5. Estimated mean shifts in upper elevation limits between the first and last survey when evaluated at the median elevation within a given region. These estimates of shifts correct for elevation by including species' initial elevation during the first survey time point as a predictor in a linear model (see Methods). Results show three additional filters as exclusion criteria for less common species (in comparison to the standard filter of >1 occurrence per species and region over all years; Fig. 3). Filters used are >5 and >10 occurrences per species and region over all years and >10 occurrences per species, region and year. Mean range shifts >0 indicate that average shifts are upslope across most of the elevational gradient, and mean range shifts at the median of the elevational gradient are considered significant when 0 lies outside of the 95% confidence interval (marked in bold). Models were not fitted in regions with fewer than 5 species after filtering.

	**>5 occurrences/ region over all years**			**>10 occurrences/ region over all years**			**>10 occurrences/ region and year**		
	**Predicted Range Shift**	**CI(95%)**	**N**	**Predicted Range Shift**	**CI(95%)**	**N**	**Predicted Range Shift**	**CI(95%)**	**N**
New South Wales, Australia	91.4	**(36.12; 146.67)**	66	90.91	**(27.1; 154.72)**	52	70.62	**(29.98; 111.25)**	24
Victoria, Australia	77.59	**(38.06; 117.13)**	55	55.3	**(23.18; 87.42)**	48	74.54	**(40.4; 108.68)**	33
Switzerland	126.91	**(3.83; 250)**	9	136.44	(-6.17; 279.06)	8			2
Central Chile	40.5	(-20.99; 101.99)	28	58.52	(-19.69; 136.74)	16			4
South Chile	93.85	**(16.79; 170.9)**	58	99.41	**(19.21; 179.61)**	50	-2.61	(-66.32; 61.11)	24
Tenerife, Spain	38.52	(-37.57; 114.61)	40	11.51	(-71.2; 94.21)	23	110. 67	(-29.61; 250.95)	6
Hawaii, USA	-113.29	**(-200.81; -25.77)**	66	-145	**(-259.92; -30.08)**	40	-131.76	(-276.41; 12.88)	10
Kashmir, India	143.8	**(50.79; 236.81)**	47	216.9	**(105.09; 328.7)**	29	170.53	**(50.04; 291.01)**	15
Montana, USA	-5.16	(-52.7; 42.38)	28	-4.18	(-57.9; 49.53)	23	-67.49	(-167.58; 32.6)	7
Norway	43.13	(-36.09; 122.36)	5			3			1
Oregon, USA	98.33	**(23.84; 172.83)**	40	69.59	**(8.03; 131.15)**	36	50.97	(-27.45; 129.39)	22

Supplementary Table 6. Relationships between species' upper elevational limit in the first survey and the change in upper elevation limit in the second survey, fitted for each region separately. Regressions were weighted by species' frequency of occurrence, tests were two-sided and no adjustments for multiple comparisons were made. Significant p-values are marked in bold.

	**Intercept ± standard error**	**Slope ± standard error**	***F***	**df**	***p***
New South Wales, Australia	241.87 ± 114.76	-0.128 ± 0.079	2.63	71	0.109
Victoria, Australia	241.21 ± 62.48	-0.153 ± 0.044	12.07	74	**0.001**
Switzerland	358.04 ± 151.34	-0.203 ± 0.116	3.08	8	0.117
Central Chile	58.29 ± 189.23	-0.008 ± 0.066	0.01	30	0.908
South Chile	364.5 ± 108.94	-0.28 ± 0.085	10.91	68	**0.002**
Tenerife, Spain	251.19 ± 91.08	-0.187 ± 0.08	5.46	55	**0.023**
Hawaii, USA	127.36 ± 97.49	-0.127 ± 0.044	8.34	106	**0.005**
Kashmir, India	408.51 ± 238.95	-0.108 ± 0.084	1.65	72	0.203
Montana, USA	842.42 ± 230.87	-0.34 ± 0.09	14.39	29	**0.001**
Norway	35.03 ± 47.63	0.021 ± 0.133	0.03	6	0.878
Oregon, USA	443.25 ± 184.89	-0.238 ± 0.111	4.6	45	**0.038**

Supplementary Table 7. Relationships between species' upper elevational limit in the first survey and the change in upper elevation limit in the second survey, fitted for each region separately. In contrast to Supplementary Table 6, regressions were not weighted by species' frequency of occurrence. Tests were two-sided and no adjustments for multiple comparisons were made. Significant p-values are marked in bold.

	**Intercept ± standard error**	**Slope ± standard error**	***F***	**df**	***p***
New South Wales, Australia	250.13 ± 124.66	-0.177 ± 0.094	3.53	71	0.064
Victoria, Australia	337.65 ± 75.49	-0.257 ± 0.06	18.6	74	**< 0.001**
Switzerland	374.03 ± 130.58	-0.222 ± 0.11	4.06	8	0.079
Central Chile	259.89 ± 273.92	-0.101 ± 0.105	0.92	30	0.345
South Chile	495.12 ± 121.97	-0.432 ± 0.111	15.2	68	**< 0.001**
Tenerife, Spain	268.27 ± 98.01	-0.206 ± 0.091	5.08	55	**0.028**
Hawaii, USA	145.79 ± 87.75	-0.143 ± 0.05	8.27	106	**0.005**
Kashmir, India	198.72 ± 191.53	-0.06 ± 0.074	0.66	72	0.419
Montana, USA	1256.23 ± 351.27	-0.516 ± 0.144	12.76	29	**0.001**
Norway	24.36 ± 38.52	0.064 ± 0.119	0.29	6	0.608
Oregon, USA	416.23 ± 173	-0.234 ± 0.115	4.18	45	**0.047**

Supplementary Table 8. Average yearly temperature change for all regions. Using CHELSA cruts monthly minimum and maximum temperature data between 2000 and 2016[1,2] downloaded for all sampling points included in the study, we fitted linear models of average annual temperature over time separately for all regions, resulting in an estimated yearly temperature change. Based on the average yearly temperature change, a total change between 2000 and 2016 was calculated. Tests were two-sided and no adjustments for multiple comparisons were made. Significant temperature changes are marked in bold.

	**Estimate ± standard error**	**Temperature increase over 17 years**	***p***	***N***
New South Wales, Australia	0.03 ± 0.02	0.45	0.146	17
Victoria, Australia	0.03 ± 0.01	0.54	0.05	17
Switzerland	0.03 ± 0.02	0.44	0.31	17
Central Chile	0.04 ± 0.02	0.63	0.061	17
South Chile	0.04 ± 0.02	0.62	0.05	17
Tenerife, Spain	0.02 ± 0.01	0.3	0.224	17
Hawaii, USA	0.02 ± 0.02	0.41	0.183	17
Kashmir, India	-0.01 ± 0.02	-0.09	0.765	17
Montana, USA	0.03 ± 0.03	0.54	0.367	17
Norway	0.03 ± 0.03	0.44	0.447	17
Oregon, USA	0.05 ± 0.03	0.89	0.077	17

Supplementary Table 9. Linear model results of the effect of plot type (disturbed plots alongside the road vs. semi-natural plots away from the road) on range shifts (elevation ± standard error), fitted separately for all regions with enough occurrence points (>1 per region over all years for both plot types) in both road and semi-natural plots and weighted by species' frequency of occurrence. Tests were two-sided and no adjustments for multiple comparisons were made. Significant differences between plot types are marked in bold (only South Chile). Across all regions combined, fitting a LMM of range limit changes on plot type (region included as a random factor, regression weighted by species' frequency of occurrence) and comparing it to an intercept only model with a likelihood ratio test revealed no significant effect of plot type (χ^2= 1.989, p < 0.159).

	**Semi-natural plot ± standard error**	**Effect roadplot ± standard error**	***F***	**df**	***p***
New South Wales, Australia	90.76 ± 53.83	-25.15 ± 57.87	0.19	93	0.665
Switzerland	-54.36 ± 91.14	162.19 ± 105.82	2.35	14	0.148
Central Chile	29.07 ± 34.93	6.86 ± 49.95	0.02	46	0.891
South Chile	-31.28 ± 26.38	85.34 ± 36.19	5.56	116	**0.02**
Tenerife, Spain	-17.9 ± 43.36	89.59 ± 54.38	2.71	84	0.103
Kashmir, India	106.55 ± 40.19	-8.64 ± 58.52	0.02	122	0.883
Montana, USA	-21.95 ± 42.64	-0.43 ± 51.85	0	48	0.993
Oregon, USA	57.19 ± 34.82	-6.03 ± 44.52	0.02	81	0.893

Supplementary Table 10. Proportions of fitted values that fall above or below the 95% confidence interval for the expected null relationship between initial upper elevation limit and change in upper elevation limit after accounting for the geometric constraint (see Fig. 1; the calculations are based on the original null model used for Fig. 4, see Methods). The deviations from null expectations of shifts were determined for both five-year intervals for all regions surveyed for a total of 10 years. Non-zero values suggest a significant deviation from the null expectation. Trends for New South Wales and Montana in years 0 to 5 are identical to Figure 4, whereas values deviating from the 95% confidence interval of the null relationship in South Chile were mainly falling above the confidence interval in the middle of the gradient and not in the lower part of the gradient as in Figure 4. Trends in New South Wales, Central Chile, South Chile and Montana in years 5 to 10 are identical to Figure 4.

	Proportion of fitted values falling outside of 95% confidence interval	
	**Years 0 to 5**	**Years 5 to 10**
New South Wales, Australia	0.633	0.54
Switzerland	0	0
Central Chile	0	0.789
South Chile	0.174	0.73
Montana, USA	0.59	0.649

Supplementary Methods: Regression Toward the Mean

Regression toward the mean (RTM) occurs whenever there is a non-perfect correlation between measurements of observational units over time and was first described by Sir Francis Galton[3]. Galton observed that unusually large or small values on average tend to be followed by measurements closer to the mean. This results in a negative correlation between the change in measurements over time and their initial state. While RTM is widely discussed in other fields, such as medicine and psychology[4,5], there has been reported to be less awareness of this phenomenon in ecology[6] (but see ref. [7]). In order to correct for RTM, Mazalla[6] proposes to always include initial measurements in analyses on temporal change. In our study, the geometric constraint of a finite environmental gradient adds another layer of of complexity to expected changes in range limits over time.

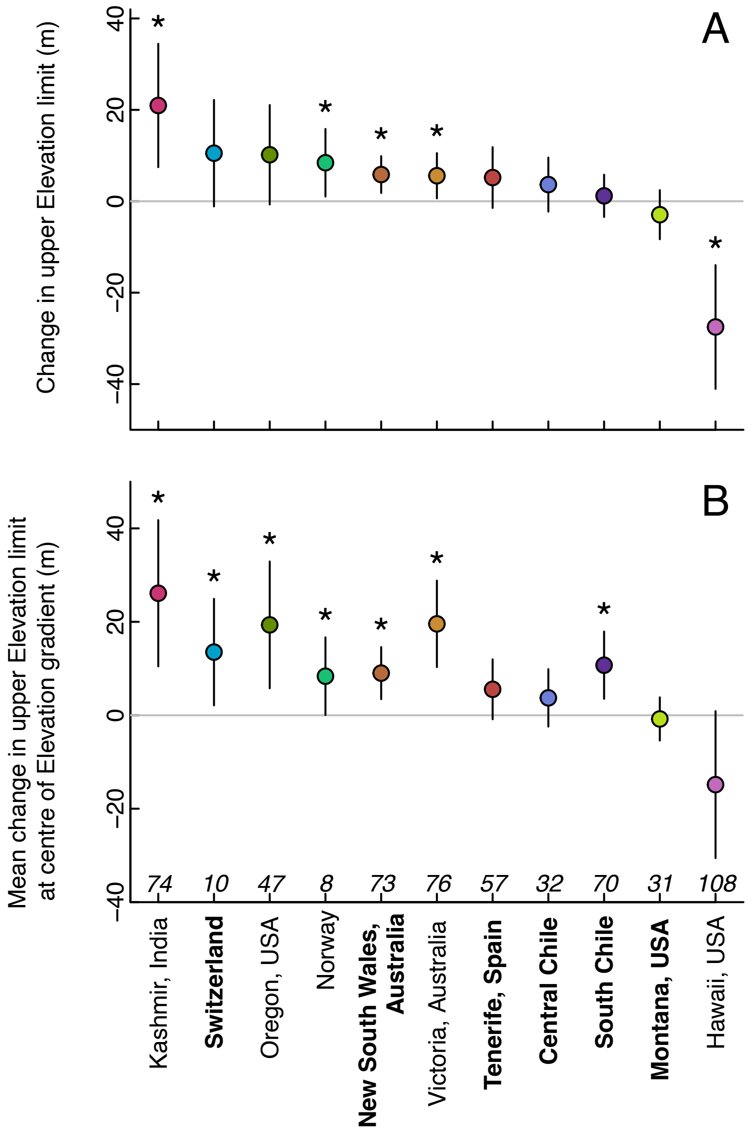

Supplementary Figure 1. Observed annual changes in species' upper elevational limits (±95% confidence intervals). Both panels show mean annual shifts, estimated from linear models that weight species by their total frequency of occurrence in both years and are fitted to data from each region separately. Panel A shows results of intercept-only models (i.e. grand mean shifts per region), while panel B shows results of models that correct for elevation by including species' initial elevation limit during the first survey as a linear predictor. Specifically, estimates in B correspond to the predicted mean shift in elevation limits when evaluated at the median elevation within a given region; values >0 therefore indicate that average shifts are upslope across most of the elevational gradient (cf. Fig. 4). Regions are ordered by effect size in panel A, with labels in regular and bold typeface indicating regions with 5- or 10-year survey intervals, respectively. Estimates that differ significantly from zero are indicated by *. Numbers in italics describe the sample size, colours correspond to the same labels as in Fig. 2

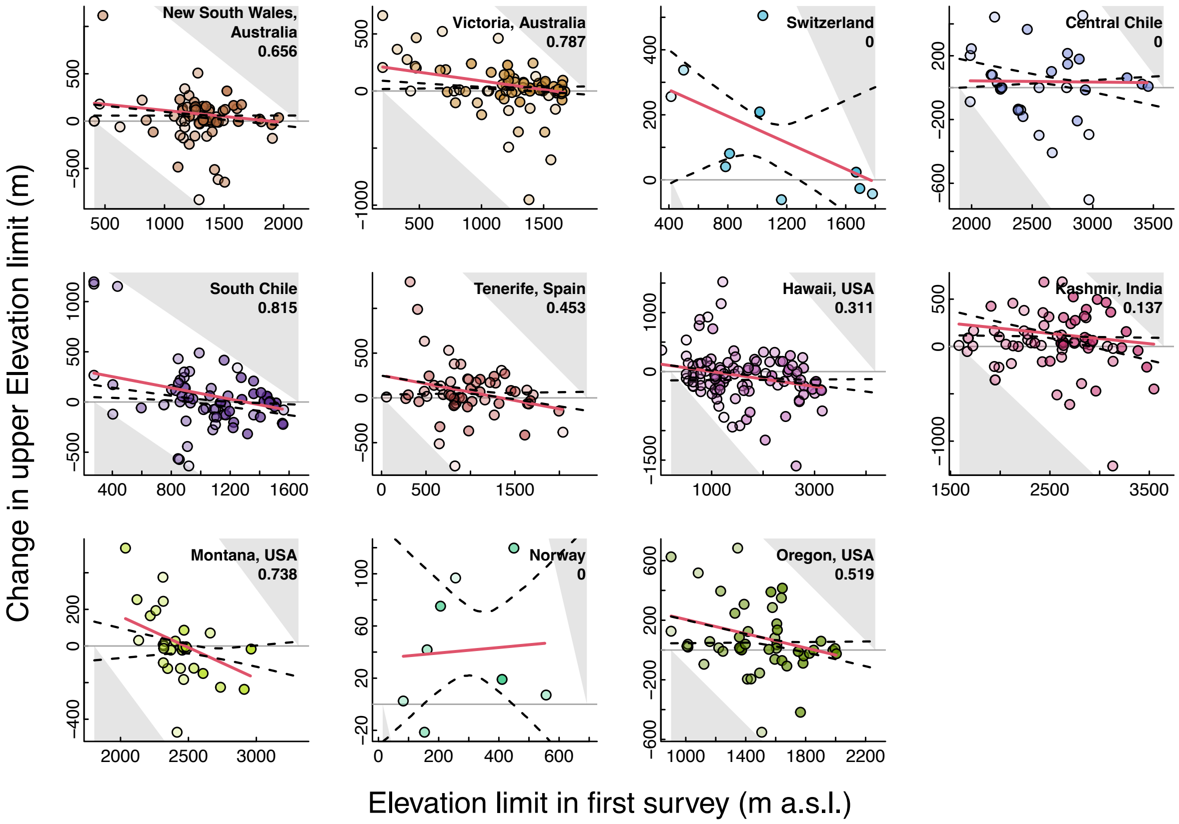

Supplementary Figure 2. Null-model tests of elevation-dependent shifts in upper elevation limit of non-native plants in 11 mountain regions. Each point shows the change in upper elevation limit (90th quantile of elevational distribution) of a single species, as a function of its limit in the first survey (darker shading corresponds to greater total log[frequency of occurrence] of a species in both surveys). Dashed lines are 95% confidence intervals for the expected null relationship between initial upper elevation limit and change in upper elevation limit after accounting for geometric constraints. This plot differs from Fig. 4 in the vector of new initial elevations used for bootstrapping, which in this case split the surveyed elevational gradient into 200 equidistant elevational bins (see Methods). Colours correspond to the same labels as in Fig. 2. For further explanation about the plot and scale for gradient scheme, see Fig. 4.

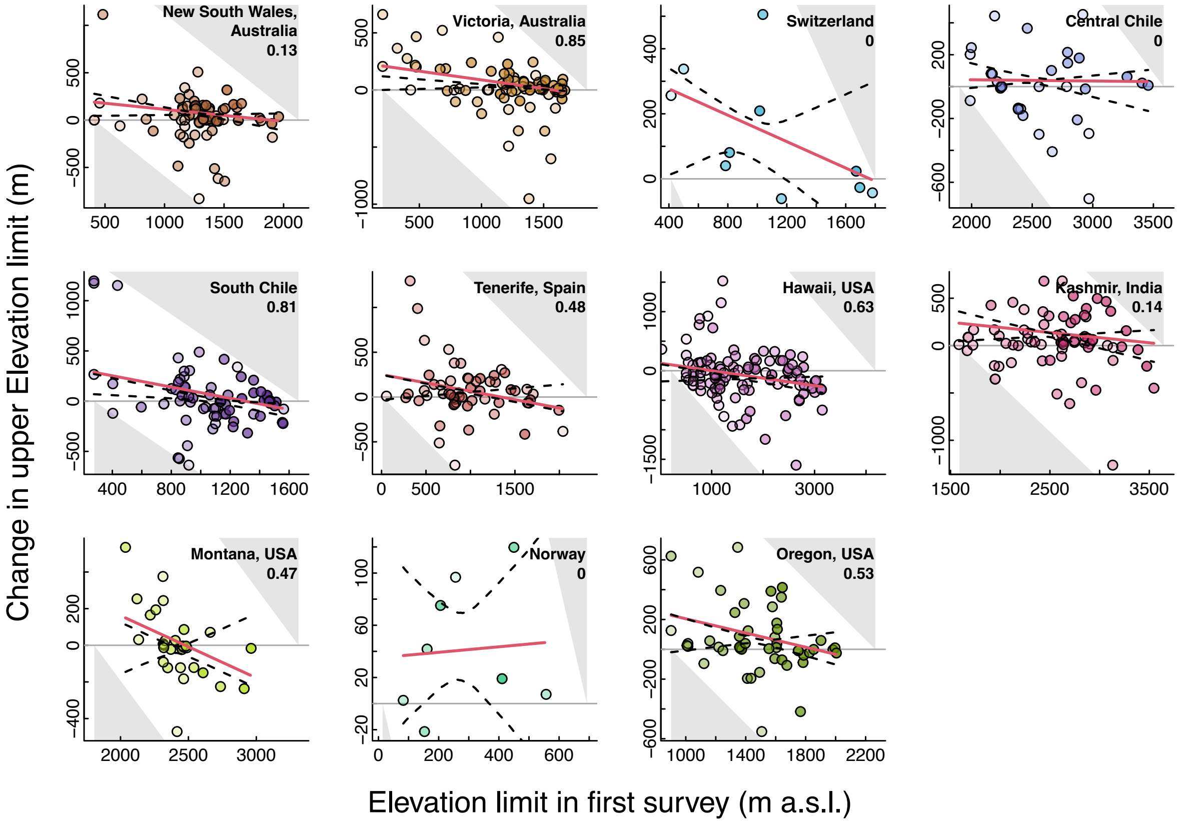

Supplementary Figure 3. Null-model tests of elevation-dependent shifts in upper elevation limit of non-native plants in 11 mountain regions. Each point shows the change in upper elevation limit (90th quantile of elevational distribution) of a single species, as a function of its limit in the first survey (darker shading corresponds to greater total log[frequency of occurrence] of a species in both surveys). Dashed lines are 95% confidence intervals for the expected null relationship between initial upper elevation limit and change in upper elevation limit after accounting for geometric constraints. This plot differs from Fig. 4 in the vector of new initial elevations used for bootstrapping, which in this case only sampled from within the vector of observed initial elevation limits (i.e. with length equal to the number of recorded species; see Methods). Colours correspond to the same labels as in Fig. 2. For further explanation about the plot and scale for gradient scheme, see Fig. 4.

Supplementary References

1 Karger, D. N. *et al.* Climatologies at high resolution for the earth's land surface areas. *Scientific Data* **4**, 170122, doi:10.1038/sdata.2017.122 (2017).

2 Karger, D. N. & Zimmermann, N. E. (EnviDat, 2018).

3 Stigler, S. M. Regression towards the mean, historically considered. *Statistical Methods in Medical Research* **6**, 103-114, doi:10.1177/096228029700600202 (1997).

4 Hannon, B. A., Thomas, D. M., Siu, C. O. & Allison, D. B. Neglecting regression to the mean continues to lead to unwarranted conclusions: Letter regarding "The magnitude of weight loss induced by metformin is independently associated with BMI at baseline in newly diagnosed type 2 diabetes: Post-hoc analysis from data of a phase IV open-labeled trial". *Adv Clin Exp Med* **28**, 1569-1570, doi:10.17219/acem/94158 (2019).

5 Medin, D., Bennis, W. & Chandler, M. Culture and the Home-Field Disadvantage. *Perspectives on Psychological Science* **5**, 708-713, doi:10.1177/1745691610388772 (2010).

6 Mazalla, L. & Diekmann, M. Regression to the mean in vegetation science. *Journal of Vegetation Science* **33**, e13117, doi:10.1111/jvs.13117 (2022).

7 Kelly, C. & Price, Trevor D. Correcting for Regression to the Mean in Behavior and Ecology. *The American Naturalist* **166**, 700-707, doi:10.1086/497402 (2005).
