# Supplementary material for: Rapid upwards spread of non-native plants in mountains across continents
Source: Nat Ecol Evol. 2023 Jan 26;7(3):405–13. doi: 10.1038/s41559-022-01979-6 (PMC9998268; doi:10.1038/s41559-022-01979-6)
Supplement: Supplementary file 2 — Reporting Summary [file 41559_2022_1979_MOESM2_ESM.pdf]

nature portfolio

Corresponding author(s): Evelin Iseli, Institute of Integrative Biology, ETH Zurich, Universitätsstrasse 16, CH-8092 Zurich, Switzerland; evelin.iseli@usys.ethz.ch

Last updated by author(s): 2022/12/08

Reporting Summary

Nature Portfolio wishes to improve the reproducibility of the work that we publish. This form provides structure for consistency and transparency in reporting. For further information on Nature Portfolio policies, see our Editorial Policies and the Editorial Policy Checklist.

Statistics

For all statistical analyses, confirm that the following items are present in the figure legend, table legend, main text, or Methods section.

n/a	Confirmed	
☐	☒	The exact sample size (*n*) for each experimental group/condition, given as a discrete number and unit of measurement
☐	☒	A statement on whether measurements were taken from distinct samples or whether the same sample was measured repeatedly
☐	☒	The statistical test(s) used AND whether they are one- or two-sided<br>*Only common tests should be described solely by name; describe more complex techniques in the Methods section.*
☐	☒	A description of all covariates tested
☐	☒	A description of any assumptions or corrections, such as tests of normality and adjustment for multiple comparisons
☐	☒	A full description of the statistical parameters including central tendency (e.g. means) or other basic estimates (e.g. regression coefficient) AND variation (e.g. standard deviation) or associated estimates of uncertainty (e.g. confidence intervals)
☐	☒	For null hypothesis testing, the test statistic (e.g. *F*, *t*, *r*) with confidence intervals, effect sizes, degrees of freedom and *P* value noted<br>*Give P values as exact values whenever suitable.*
☒	☐	For Bayesian analysis, information on the choice of priors and Markov chain Monte Carlo settings
☐	☒	For hierarchical and complex designs, identification of the appropriate level for tests and full reporting of outcomes
☐	☒	Estimates of effect sizes (e.g. Cohen's *d*, Pearson's *r*), indicating how they were calculated

Our web collection on statistics for biologists contains articles on many of the points above.

Software and code

Policy information about availability of computer code

Data collection	No code was used to collect the data. Identity of nonnative vascular plants was defined according to the World Flora Online (WFO. World Flora Online, <http://www.worldfloraonline.org> (2019)). Taxonomic harmonization to detect synonyms for the same species in different regions and to correct spelling problems was done by the MIREN data managers, using the R-packages "taxize" and "WorldFlora".
Data analysis	All analyses were carried out in R, version 4.0.3, using the "lme4" package. The code produced for the analysis is available as a supplementary file.

For manuscripts utilizing custom algorithms or software that are central to the research but not yet described in published literature, software must be made available to editors and reviewers. We strongly encourage code deposition in a community repository (e.g. GitHub). See the Nature Portfolio guidelines for submitting code & software for further information.

Data

Policy information about availability of data

All manuscripts must include a data availability statement. This statement should provide the following information, where applicable:

- Accession codes, unique identifiers, or web links for publicly available datasets
- A description of any restrictions on data availability
- For clinical datasets or third party data, please ensure that the statement adheres to our policy

All datasets generated before 2016 (except two roads in Victoria, Australia) and analysed during the current study are available through the Global Biodiversity Information Facility (G Bl F, https://www.gbif.org/publisher /76388ab6-61ca-439a-ab09-e 1 fe 73eb224a) and the complete data set is available on Zenodo (https:// doi .org/10.5281/zenodo.5529072). Environmental data and a summary file containing all species with their respective range shifts are provided as a supplementary files.

Field-specific reporting

Please select the one below that is the best fit for your research. If you are not sure, read the appropriate sections before making your selection.

☐ Life sciences ☐ Behavioural & social sciences ☒ Ecological, evolutionary & environmental sciences

For a reference copy of the document with all sections, see nature.com/documents/nr-reporting-summary-flat.pdf

Ecological, evolutionary & environmental sciences study design

All studies must disclose on these points even when the disclosure is negative.

Study description

The study seeks to monitor non-native plant species richness and abundance along elevational gradients in mountainous regions around the world. It is based on data collected by the Mountain Invasion Research Network (MIREN) using a standard protocol replicated across 11 mountainous regions around the world: the Australian Alps (two regions, New South Wales and Victoria); the Swiss Alps; the Andes (two regions, Central and South Chile); the Montana-Yellowstone National Park (USA); the Blue Mountains (Oregon, USA); Hawaii (USA); Tenerife (Canary Islands, Spain); Kashmir (India); and the Northern Scandes (Norway). To ensure within-region replication, three roads were monitored in each region (two in Central Chile, four in Hawaii, five in Victoria), resulting in a spatially nested data set. Sampling was repeated during peak growing season at 5-year intervals, starting in 2007 and ending in 2017 (sampling period and frequency differs between regions, see "Timing and spatial scale").

Research sample

The research sample consists of occurrence points of all nonnative plant species and individuals recorded in the sampling locations described below.

Sampling strategy

Each monitored road was evenly stratified by elevation into 20 sampling locations (i.e., 60 per region, though this varied due to local logistics), totaling 651 sampling locations. At each location, three 2 x 50 m plots were placed in a T-shape (i.e., up to 50 and 100 m away from road verges). The two perpendicular plots were only surveyed when there were no impassable barriers such as cliffs and rivers, resulting in unequal numbers of plots per sampling location (respectively 655, 487 and 445 plots at 0, 50 and 100 m from the road). In every plot, the identity of nonnative vascular plants according to the World Flora Online and their abundance (scale 1 = 1-10 individuals (or ramets), 2 = 11-100 individuals and 3 > 100 individuals) was recorded.
Sample size is determined by the number of participating regions, accessible plots and nonnative species present.

Data collection

The data was collected individually in all regions by the responsible MIREN members according to a standard protocol (Haider, S. et al. Think globally, measure locally: The MIREN standardized protocol for monitoring species distributions along elevation gradients. Ecology and Evolution 12, e8590, doi: 10.1002/ece3.8590 (2021).).

Timing and spatial scale

Sampling years and geolocation (in brackets) for all regions:

New South Wales, Australia: 2007 / 2012/ 2017 (-36.038, 148.359)
Victoria, Australia: 2012/ 2017 (-37.169, 147.075)
Swiss Alps: 2007/ 2012/ 2017 (46.261, 7.503)
Central Chile: 2007/ 2012/ 2017 (-33.343, -70.28)
South Chile: 2007/ 2012/ 2017 (-37.564, -71.568)
Montana-Yellowstone National Park, USA: 2007/ 2012/ 2017 (44.777, -110.196)
Blue Mountains, Oregon, USA: 2007/ 2012 (45.235, -117.531)
Hawaii, USA: 2007 / 2012 (19.981, -155.657)
Tenerife, Canary Islands, Spain: 2008/ 2018 (28.254, -16.604)
Kashmir, India: 2012/ 2017 (33.622, 74.985)
Northern Scandes, Norway: 2012/ 2017 (68.248, 17.651)

Sampling was repeated during peak growing season. A 5-year interval between sampling seasons is chosen to promote long-term monitoring .

Data exclusions

Species not keyed to the species level plus species occurring only once in a region were excluded from the analysis. The frequency cut-off was defined to reduce possible bias caused by very infrequent species. This exclusion criteria was defined at the beginning of the analysis. An additional sensitivity analysis with stricter exclusion criteria is described in the manuscript.

Reproducibility

Reproducibility of data collecting is achieved by a standard sampling protocol applied in all participating regions (see "Data collection").

Randomization

To account for the spatial nestedness of the data, regional data was either analyzed separately or region was included as a random effect in linear mixed models.

Blinding

Data were observational data collected in the field and so blinding with respect to location/elevation could not be performed. For re-surveys, lists of species found in previous visits to a plot were available but not reviewed until after data collection in a given plot, if at all. Since there were no experimental treatments, blinding was not relevant during data analysis.

Did the study involve field work? ☒ Yes ☐ No

Field work, collection and transport

Field conditions	Field conditions differed between regions and years and specific information is not included in the combined available data set.
Location	Geolocation of all sampled regions is stated in "Timing and spatial scale". Elevational range (m.a.s.l.) within regions:<br><br>New South Wales, Australia: 410-2125<br>Victoria, Australia: 205 - 1848<br>Swiss Alps: 415-1800<br>Central Chile: 1900 - 3585<br>South Chile: 277 - 1664<br>Montana-Yellowstone National Park, USA: 1807-3311<br>Blue Mountains, Oregon, USA: 902-2264<br>Hawaii, USA: 212 - 4180<br>Tenerife, Canary Islands, Spain: 13-2310<br>Kashmir, India: 1590-3644<br>Northern Scandes, Norway: 14 - 692
Access & import/export	Where permits to access sampling locations were needed (e.g. national parks), they were obtained by the responsible authorities in advance to the field work. As the collected data consists of plant richness and abundance data and therefore no samples have been taken, no import/ export permits were required.<br><br>Permits issued in the following regions:<br>- Montana-Yellowstone National Park, USA: permits issued from Yellowstone National Park for two of the three surveyed roads, starting in 2006<br>- Tenerife, Canary Islands, Spain: permit issued by Cabildo de Tenerife in 2008 and 2018
Disturbance	The disturbance by the study was minimal, as no samples were taken and no treatments were executed. The only disturbance was the permanent marking of all plot corners with magnets or metal tags.

Reporting for specific materials, systems and methods

We require information from authors about some types of materials, experimental systems and methods used in many studies. Here, indicate whether each material, system or method listed is relevant to your study. If you are not sure if a list item applies to your research, read the appropriate section before selecting a response.

Materials & experimental systems

n/a	Involved in the study	
☒	☐	Antibodies
☒	☐	Eukaryotic cell lines
☒	☐	Palaeontology and archaeology
☒	☐	Animals and other organisms
☒	☐	Human research participants
☒	☐	Clinical data
☒	☐	Dual use research of concern

Methods

n/a	Involved in the study	
☒	☐	ChIP-seq
☒	☐	Flow cytometry
☒	☐	MRI-based neuroimaging
